# Tensile and Low-Cycle Fatigue Behavior, Fracture Mechanisms, and Life Predictions of 316H Stainless Steel at 600~800 °C

**DOI:** 10.3390/ma19061228

**Published:** 2026-03-20

**Authors:** Xiaoyang Sun, Zhengxin Tang, Xikou He

**Affiliations:** 1 Institute for Special Steels, Central Iron and Steel Research Institute Co., Ltd., Beijing 100081, China; sunxiaoyangcn@163.com (X.S.); tangzhengxin@nercast.com (Z.T.); 2 State Key Laboratory of Advanced Special Steel, Beijing 100081, China; 3 NCS Testing Technology Co., Ltd., Beijing 100081, China

**Keywords:** 316H stainless steel, low cycle fatigue, fracture mechanisms, fatigue life prediction

## Abstract

In this study, the tensile properties, low-cycle fatigue behavior, and microscopic fatigue-failure mechanisms of 316H stainless steel in the temperature range of 600–800 °C were systematically investigated by means of tensile tests, high-temperature low-cycle fatigue tests, and scanning electron microscopy (SEM) analysis of fatigue fracture surfaces. Based on experimental data fitting, a life prediction model for the material in the high-temperature regime was established. The results indicate that the mechanical behavior of 316H stainless steel under both static and cyclic loading is significantly influenced by temperature and strain amplitude. Compared with its room-temperature properties, at 800 °C, the elastic modulus of 316H stainless steel decreases by approximately 30%, the tensile strength drops by about 60%, while the elongation after fracture increases by roughly 100%. Within the temperature range of 600–800 °C, the fatigue performance deteriorates with the increasing temperature, and the cyclic hardening rate accelerates as the temperature rises. The fracture mode in the instantaneous fracture zone of the fatigue fracture surface transitions from predominantly transgranular fracture to a mixed mode of transgranular and intergranular fracture as the temperature increases to 800 °C. Under higher strain amplitudes (around 0.6%), 316H stainless steel exhibits Masing behavior and dynamic strain aging (DSA). Correspondingly, the crack-initiation mode on the fatigue fracture surface shifts from a single surface source to multiple surface sources. A three-parameter model was employed to fit the strain–amplitude versus fatigue–life relationships of 316H stainless steel in the 600–800 °C range, showing good agreement with the experimental data, with most data points falling within a factor-of-two error band.

## 1. Introduction

For reactor core support materials and pipeline components, prolonged high-temperature operation in nuclear power plants exposes these materials to cyclic thermal transients, equipment start-up/shutdown cycles, and localized stress concentration within structural elements. Such operational conditions inevitably induce material degradation mechanisms including creep deformation, fatigue damage, and their synergistic creep–fatigue interaction. During the start-up and shutdown cycles of power generation units, the temperature gradients generated subject the materials to cyclic thermal stresses. Consequently, low-cycle fatigue (LCF) is recognized as one of the primary damage mechanisms affecting the structural integrity of reactor pressure vessels and piping systems [1]. Therefore, in the design of power plants, low-cycle fatigue performance requirements are typically specified for these materials [2].

The Generation IV nuclear reactors are designed with operational temperature ranges from 500~800 °C [3,4,5,6,7,8,9]. To meet the performance requirements under such conditions, the United States and Russia have adopted the strategy of elevating carbon content in austenitic stainless steels to enhance their elevated-temperature mechanical properties [8]. Consequently, 316H stainless steel has been developed and is extensively utilized in reactor core components and other nuclear pressure vessel applications due to its superior high-temperature strength and enhanced creep resistance.

Due to its higher carbon content compared to 316 stainless steel, 316H austenitic stainless steel exhibits superior high-temperature resistance. This makes it particularly suitable for nuclear piping systems that transport radioactive fluids. Additionally, it does not undergo brittle failure at low temperatures, rendering it an ideal material for nuclear reactor applications [9]. The 316H stainless steel exhibits a maximum service temperature of 800 °C, as specified in the ASME Boiler and Pressure Vessel Code [10], demonstrating exceptional thermal stability under prolonged high-temperature exposure. This material is recognized as a promising candidate material for Generation IV reactor systems. Jingyu Yang et al. [11] systematically investigated the correlation between the low-cycle fatigue (LCF) strain amplitude and dislocation configuration evolution in 316H stainless steel at 650 °C. Through cyclic stress decomposition methodology, the researchers quantitatively characterized stress saturation phenomena and established microstructure-based fracture mechanics models to interpret fracture mechanisms. Furthermore, Xu et al. [12] examined the strain amplitude dependence of LCF behavior in 316H stainless steel at 550 °C. Their work focused on establishing relationships between microstructural evolution and cyclic stress response characteristics. Subsequently, Zhao et al. [13] conducted a study on the fatigue performance of 316H stainless steel with varying grain sizes under ambient temperature conditions. Their findings revealed that the microstructural characteristics of dislocation exhibited pronounced grain size dependency.

Based on the above considerations, the low-cycle fatigue performance at 600–800 °C constitutes a critical design parameter for assessing whether 316H stainless steel can be used as a structural material in Gen-IV reactors. Current research on 316H stainless steel generally focused on the temperature range of 550–650 °C. However, systematic studies of 316H steel above 650 °C remain limited. Therefore, this work systematically investigates the fatigue behavior and fracture mechanisms of 316H stainless steel in the temperature interval of 600–800 °C and develops a suitable low-cycle fatigue life prediction model. The study aims to provide essential experimental data and theoretical support for the potential application of 316H stainless steel in Gen-IV nuclear reactors.

## 2. Materials and Methods

The experimental material in this study was industrially produced 316H stainless steel forgings by Fushun Special Steel Co., Ltd., Fushun, China. The manufacturing process comprised the following steps: raw material selection, electric arc furnace primary smelting, electroslag remelting, ingot blooming, forging, annealing, and surface descaling to obtain a Φ350 mm bar by forging. Subsequently, the bar was subjected to solution at 1050 ± 5 °C for 40 min, followed by water quenching, complying with the requirements specified in ASME SA-182/SA-182M [14]. Prior to mechanical testing, the chemical composition of the forged bar was quantitatively analyzed in accordance with GB/T 223 [15] series standards. The element composition is summarized in Table 1.

**Figure 1:**
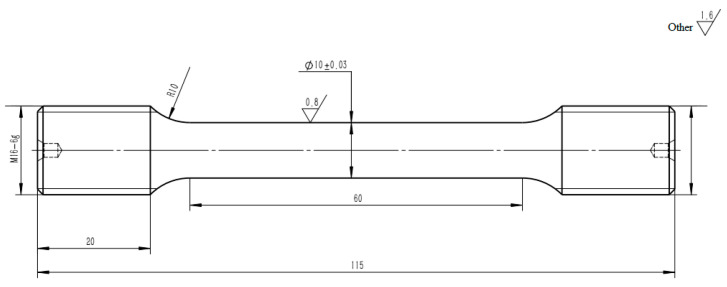
Geometry of tensile specimen.

The forging bar is processed into a tensile specimen with a diameter of Φ10 mm; the geometry of the specimen is shown in Figure 1.

Uniaxial tensile tests were conducted at ambient temperature (25 °C), 600 °C, 650 °C, and 800 °C, following the specifications of GB/T 228.1-2010 (Metallic Materials—Tensile Testing at Elevated Temperatures: Part 1) [16] and GB/T 228.2-2015 (Metallic Materials—Tensile Testing at Elevated Temperatures: Part 2) [17]. The specimens were loaded under constant stain rate conditions, which are 1.0 × 10^−4^/ s before yielding and 6.7 × 10^−3^ /s after total strain exceeding 5% until fracture.

The elastic modulus and Poisson’s ratio were determined by dynamic method [18]. Measurements were performed from ambient conditions (25 °C) to elevated temperatures of 100 °C, 200 °C, 300 °C, 400 °C, 500 °C, 600 °C, 700 °C, and 800 °C. Rectangular plate specimens with nominal dimensions of 80 mm × 20 mm × 3 mm were machined to ensure geometric uniformity, axial straightness, and surface integrity. Critical surface tolerances included relative parallelism deviation of <20 μm and mean surface roughness (Ra) ≤ 1.6 μm. All tests were conducted using an RFDA-HTVP1750-C high-temperature modulus analyzer (IMCE, Genk, Belgium).

**Figure 2:**
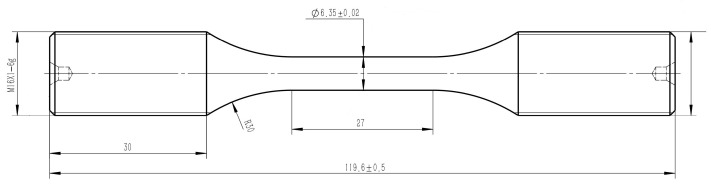
Geometry of low-cycle fatigue specimen.

Low-cycle fatigue (LCF) specimens with a nominal diameter of 6.35 mm were machined for high-temperature testing. The geometry of the specimens is illustrated in Figure 2. The specimen is designed according to GB/T 15248-2008 [19], considering extensometer clip and transverse bending. The specimens were machined into a dog-bone shape. The gauge section was sequentially processed through turning (rough and finish), followed by grinding (rough and finish), resulting in a smooth, cylindrical geometry with excellent parallelism.

**Figure 3:**
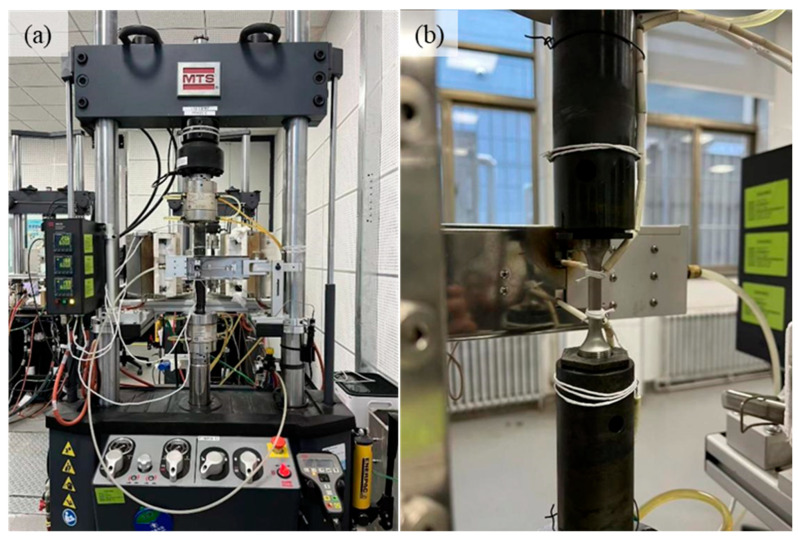
(**a**) Low-cycle fatigue equipment and (**b**) details of LCF specimen.

LCF tests were conducted at elevated temperatures of 600 °C, 650 °C, and 800 °C. At each temperature, five distinct strain amplitudes were applied, with triplicate specimens tested per strain level to ensure statistical reliability. The experiments were performed under a constantly reversed strain-controlled mode (strain ratio R = −1) at a constant strain rate of 4.0 × 10^−3^ /s. A servo-hydraulic fatigue testing system produced by MTS Systems Corporation, Eden Prairie, MN, USA (MTS Landmark series, ±100 kN load range) equipped with a high-temperature extensometer (MTS 632.53F-11 series, 25 mm gauge length) was utilized for cyclic loading. All procedures strictly adhered to GB/T 15248-2008 (Metallic Materials—Axial Force-Controlled Fatigue Testing Method) [19]. The fatigue testing system is demonstrated in Figure 3.

**Figure 4:**
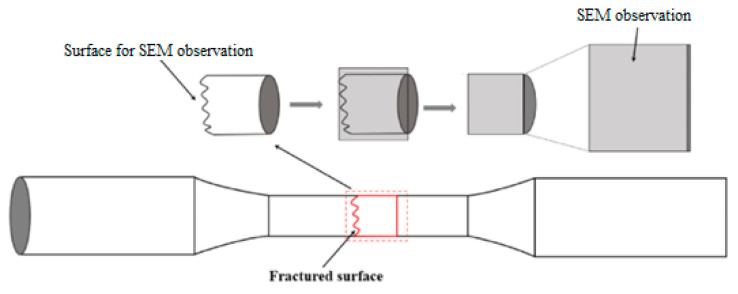
Schematic illustration of the sample preparations for SEM observations.

Fractographic and microstructural analyses were performed using a JSM-IT800 scanning electron microscope (JEOL Ltd., Akishima, Tokyo, Japan). For microstructure characterization, cross-sectional specimens were extracted from regions adjacent to the fracture surface and mechanically polished to a mirror finish. The chemical etchant was prepared by dissolving 5 g FeCl_3_ in 100 mL deionized water, followed by the addition of 20 mL concentrated hydrochloric acid (HCl, 35–38% by volume) with thorough stirring. Figure 4 schematically illustrates the way of preparing samples for SEM observations.

## 3. Results and Discussion

### 3.1. Tensile

**Figure 5:**
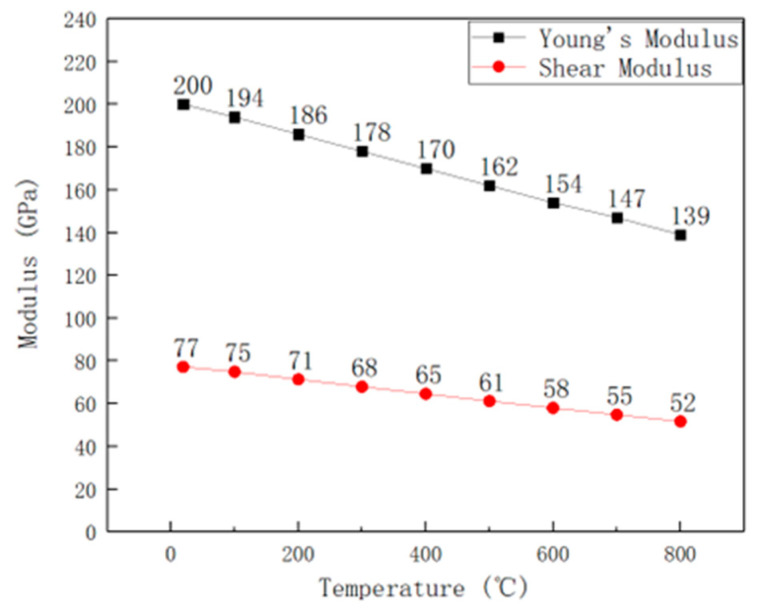
Influence of temperature on Young’s modulus and shear modulus of 316H stainless steel [20].

The temperature-dependent evolution of Young’s modulus and shear modulus from ambient temperature to 800 °C is graphically summarized in Figure 5 [20]. A linear reduction trend was observed for both moduli with increasing temperature. Quantitative analysis revealed that Young’s modulus at 800 °C decreased by 30% compared to its room-temperature value, while the shear modulus exhibited a 32% reduction under the same thermal conditions. This phenomenon is attributed to thermal expansion-induced alterations in interatomic distances within the crystal lattice, which reduces elastic stiffness at elevated temperatures [21,22,23,24,25,26,27]. The temperature–elastic modulus relationship of pure iron and the linear reduction trend of 316H stainless steel below 800 °C align closely [22]. The elastic modulus behavior of pure iron and 316H stainless steel are stable within this temperature range.

**Figure 6:**
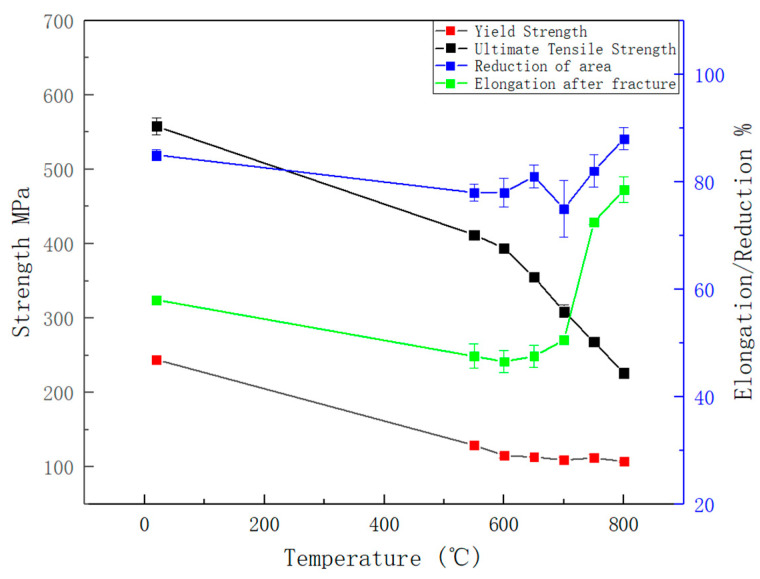
Tensile properties of 316H steel at different temperatures.

The tensile properties of 316H stainless steel across a temperature range of 25 °C to 800 °C are summarized in Figure 6. As evidenced by the data, the ultimate tensile strength (UTS) undergoes a significant reduction (50%) at a temperature range of 600–800 °C, whereas the yield strength (YS) displays minimal sensitivity (10%) to thermal variations. The strength at 800 °C decreased by approximately 64% compared to that at room temperature. In contrast, the elongation after fracture and the reduction in area, which represent key plastic indicators, exhibited an initial decrease followed by an increase with the rising temperature. Notably, at 800 °C, the elongation after fracture showed an increase of up to 100% compared to the bottom values observed at 600 °C. This phenomenon strongly suggests a significant ductility increasing at temperatures above 600 °C. The tensile fracture microstructure exhibited a substantial number of voids at 800 °C, indicating a pronounced vacancy diffusion-dominated failure mechanism at this temperature. According to the theory of dislocation motion at elevated temperatures, under the combined effect of external stress and thermal activation, dislocations overcome internal obstacles within the material and initiate slip. As the temperature increases, the thermal activation process becomes more pronounced, thereby reducing the external stress required to overcome these obstacles and consequently lowering the flow stress accordingly [28].

**Figure 7:**
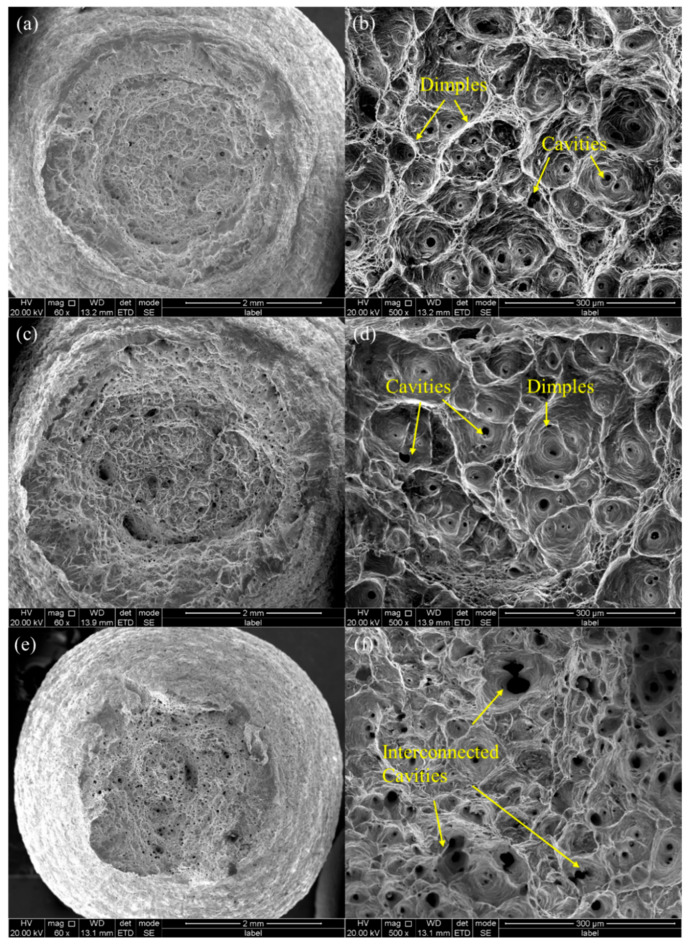
Fracture morphology of 316H steel at elevated temperature. (**a**,**b**) Room temperature tensile fracture, (**c**,**d**) 600 °C tensile fracture, and (**e**,**f**) 800 °C tensile fracture.

Fractographic analysis of 316H stainless steel under tensile loading is illustrated in Figure 7. It reveals that at a temperature range of 600–800 °C, the specimens exhibit a cup-and-cone fracture morphology with pronounced plastic deformation, consistent with ductile failure mechanisms. The fracture surface at 800 °C exhibits the minimum surface area among all tested conditions This corresponds to the significantly enhanced plastic properties observed at 800 °C. Microstructure examination further demonstrates that high-temperature fracture surfaces are dominated by dimple structures. At 600 °C and 650 °C, individual microcavities are observed at the bottom of dimples, whereas at 800 °C, the dimple becomes smaller with enlarged and interconnected cavities at their bottoms. This indicates that, during tensile deformation, the elevated temperature of 800 °C leads to a very rapid vacancy diffusion and pore formation within the material. Comparative analysis of the fracture morphologies at 600 °C, 650 °C, and 800 °C reveals a significant decrease in dimple size at 800 °C. This may be attributed to the sharp increase in the reduction in area.

### 3.2. Fatigue Behavior

**Figure 8:**
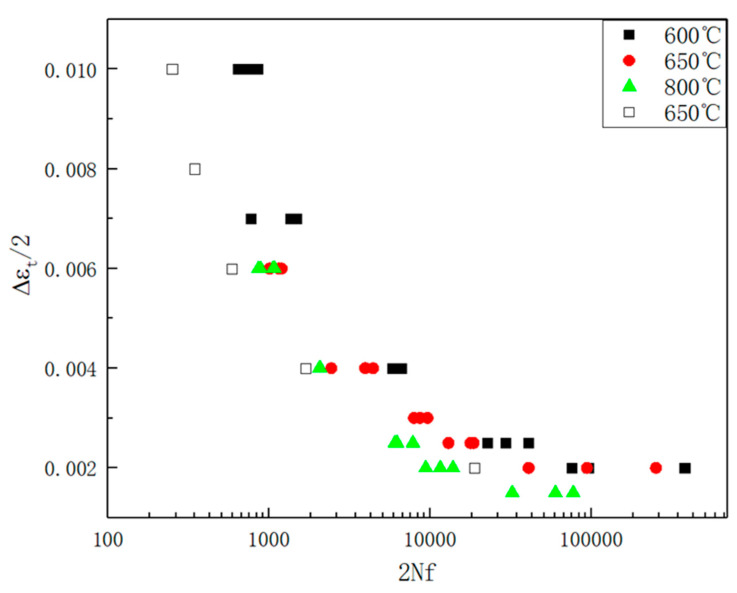
Strain-2*N_f_* curve of 316H stainless steel at different temperatures (R = −1) [11,20].

The total strain amplitude versus the number of reversals to failure (2*N_f_*) for 316H stainless steel at 600 °C, 650 °C, and 800 °C is summarized in Table 2. The strain amplitude in low-cycle fatigue tests ranges from 0.2% to 1.0%, with fatigue life spanning from 10^2^ to 10^5^ cycles, effectively covering the entire spectrum of low-cycle fatigue life. To provide an intuitive representation of the relationship between fatigue life and strain amplitude, the data in Table 2 is plotted in Figure 8. It can be observed that fatigue life is jointly influenced by the temperature and stress amplitude. With increasing temperature from 600 °C to 800 °C, the strain amplitude-2*N_f_* curves move leftward and downward, which indicate a progressive reduction in fatigue resistance under elevated thermal conditions. As stress increases, fatigue life decreases due to accelerated damage accumulation. The relationship between fatigue life and the microstructure will be discussed in the subsequent section. Furthermore, the scatter in fatigue life data becomes more pronounced with decreasing total strain amplitude. According to the general trend observed in ε-N curves, the strain amplitude exhibits an essentially linear relationship with log(2*N_f_*) [19]. Consequently, at lower strain amplitudes, the strain–life curve becomes notably flatter, where even minor deviations in the strain amplitude may lead to substantial variations in fatigue life.

**Figure 9:**
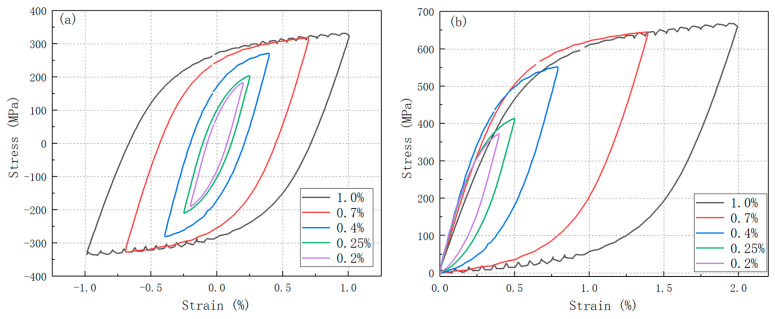

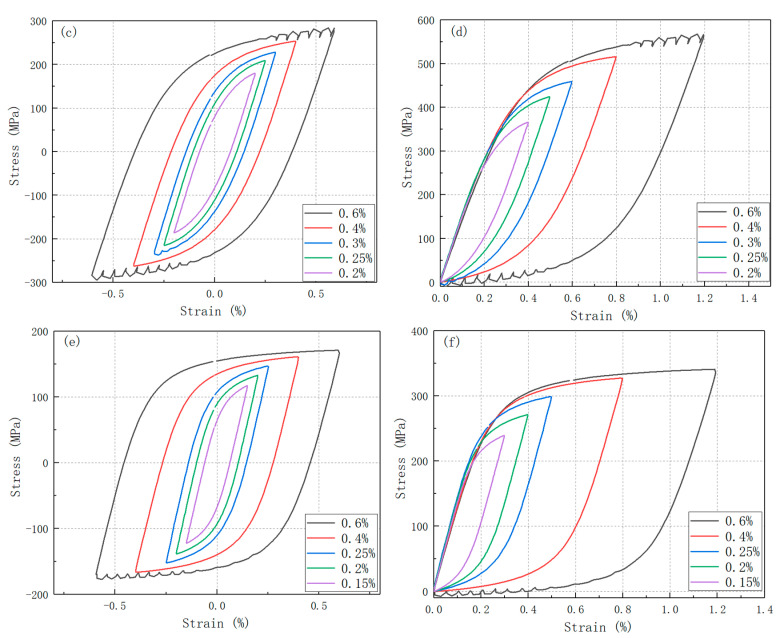
Half-life hysteresis loops of 316H stainless steel at different temperatures: (**a**,**b**) 600 °C, (**c**,**d**) 650 °C, and (**e**,**f**) 800 °C.

The hysteresis loops at the median fatigue life for various strain amplitudes at 600 °C were plotted in Figure 9. By translating the negative apex of each loop to the origin, it is evident that the material exhibits non-Masing behavior at strain amplitudes below 0.7%, transitioning to Masing behavior above this threshold strain amplitude. At 650 °C, the critical strain amplitude for Masing behavior is higher than 0.6%, whereas at 800 °C, the onset of Masing behavior is approached at 0.6% strain amplitude. Notably, the cyclic stress–strain curve at 600 °C under a strain amplitude of 1.0% reveals pronounced Portevin–Le Chatelier (PLC) effects, indicative of dynamic strain aging (DSA) within the microstructure under larger strain conditions. However, at 650 °C and 800 °C, DSA phenomena emerge at lower strain amplitudes, which was 0.6%. DSA phenomena are generally attributed to the interaction between solution atoms and dislocations [29,30,31,32,33,34]. This indicates that the degree of interaction between solute atoms and dislocations at the microscale intensifies with the increasing fatigue strain amplitude, a point that will be further elaborated in subsequent sections.

**Figure 10:**
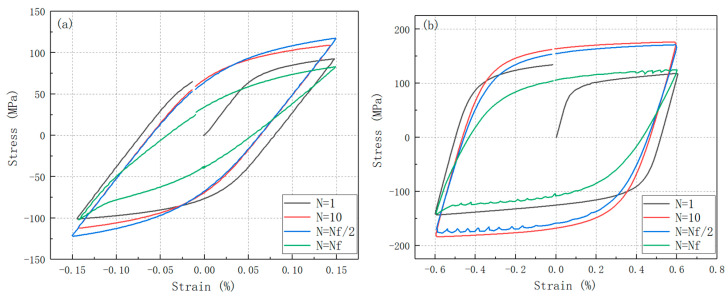
(**a**) Half-life hysteresis loops at 800 °C with 0.15% stain amplitude; (**b**) half-life hysteresis loops at 800 °C with 0.6% stain amplitude.

Hysteresis loops at various cycle numbers under constant strain amplitudes are presented in Figure 10. Figure 10a,b depict the hysteresis loops for specimens subjected to strain amplitudes of 0.15% and 0.6%, respectively, at the first cycle, tenth cycle, mid-life cycle, and final cycle. Under a strain ratio of *R* = −1, the hysteresis loops exhibit a nearly symmetrical distribution about the origin. As the strain amplitude increases, the area enclosed by the hysteresis loops expands, reflecting a corresponding rise in energy dissipation during fatigue loading [35,36]. Notably, at higher strain amplitudes, dynamic strain aging (DSA) phenomena are observed in the mid-life and final cycles, whereas no DSA effects are detected in the initial and 10th cycles. This behavior suggests that the dislocation density progressively increases with cyclic loading, eventually reaching a threshold where interactions with solution atoms become significant, thereby triggering DSA.

**Figure 11:**
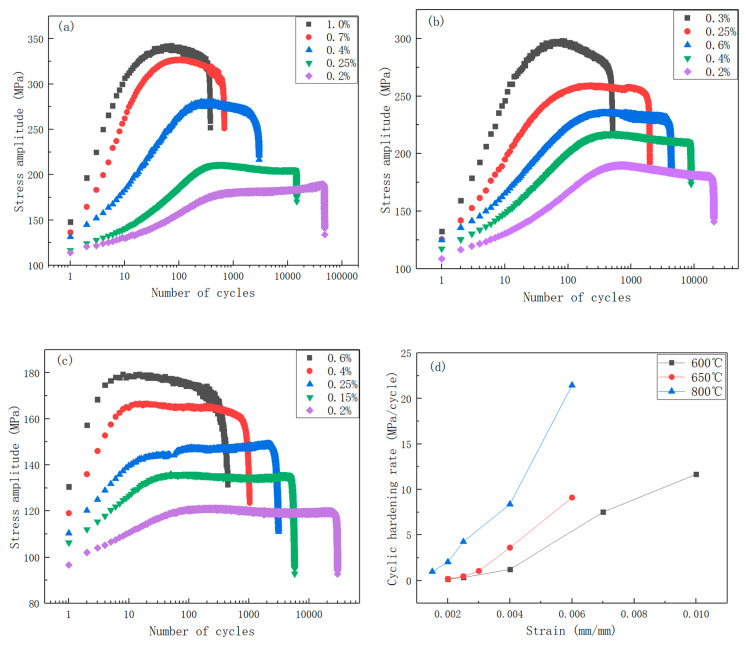
(**a**) Cyclic stress response at various strain amplitudes at 600 °C. (**b**) Cyclic stress response at various strain amplitudes at 650 °C. (**c**) Cyclic stress response at various strain amplitudes at 800 °C. (**d**) The cyclic hardening rate versus strain relationship curves at 600 °C~800 °C.

The cyclic stress response curves at half-life for various strain amplitudes and testing temperatures are illustrated in Figure 11. The stress evolution can be categorized into three stages: first stage, an initial cyclic hardening stage, characterized by a progressive increase in the stress amplitude; second stage, a subsequent cyclic stabilization stage, where the stress amplitude remains relatively constant; and the third stage, a final stage marked by crack initiation and rapid stress degradation until specimen fracture.

From the perspective of stress amplitude, a reduction in the stress amplitude is observed for a given strain amplitude as the temperature increases. It can be concluded that the interaction between dislocations and solute atoms also intensifies with an increasing number of cycles.

As illustrated in Figure 11, the cyclic hardening stage at 600 °C and 650 °C is prolonged, with the stress exhibiting a prolonged cyclic hardening stage, ranging between 50 and 300 cycles depending on the strain amplitude. However, at 800 °C, the number of cycles required to achieve cyclic stability is significantly reduced, ranging from 5 to 100 cycles across different strain amplitudes. At a specific temperature, a higher strain amplitude leads to a shorter number of cycles required to reach cyclic stability. The cyclic hardening rate, defined as the ratio of the increase in stress amplitude to the increase in the number of cycles prior to stabilization, is summarized for 316H stainless steel at various temperatures in Table 3 and Figure 11d. The cyclic hardening rate increases with both higher temperatures and larger strain amplitudes. The cyclic hardening rate was fitted using the function y = ax^b^. The fitting results, as presented in Table 4, indicate that the cyclic hardening rate essentially follows a power-law increase with the rising strain.

### 3.3. Fracture and Microstructure Observations

**Figure 12:**
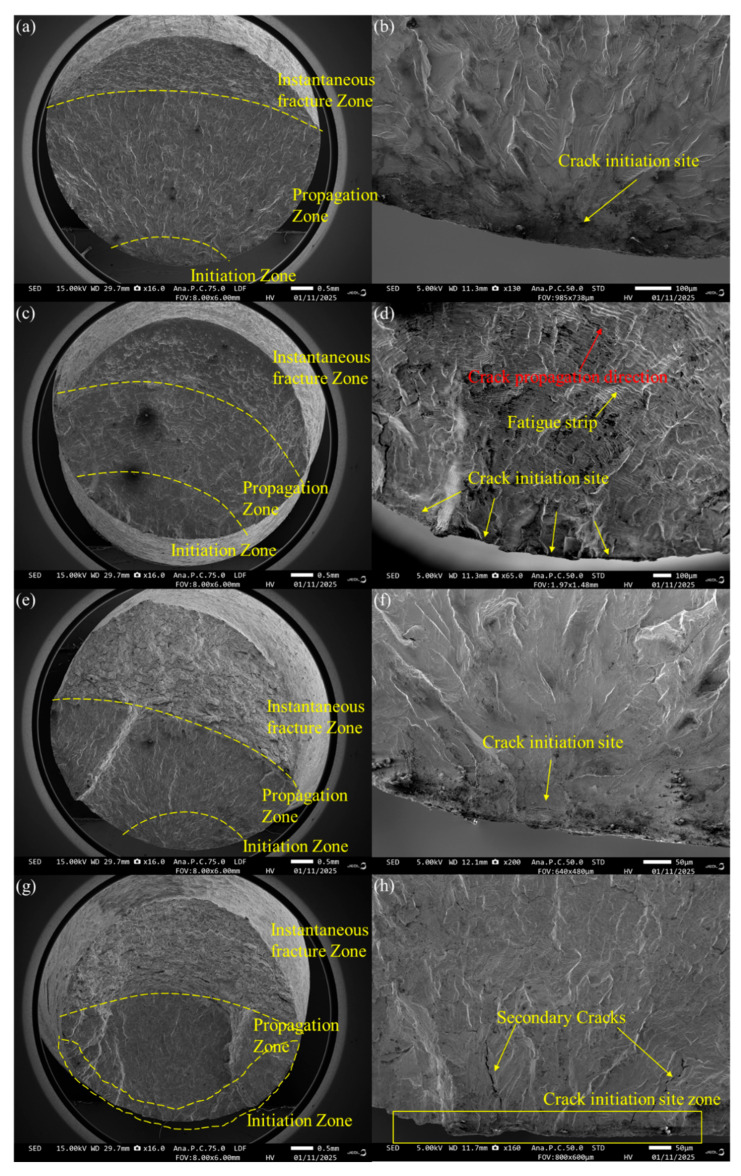

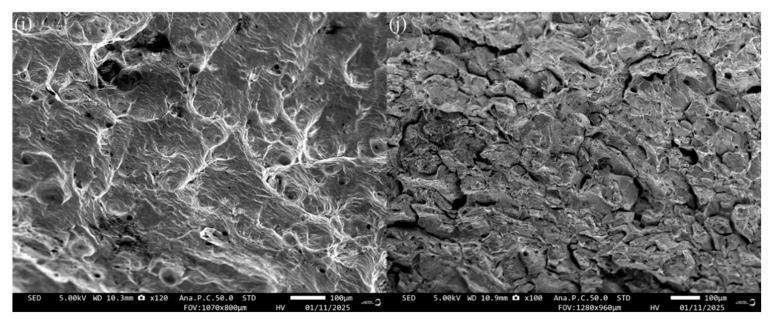
Morphologies of fracture surface for 316H stainless steel after fatigue test under high temperatures: (**a**,**b**) 0.2% strain amplitude at 600 °C; (**c**,**d**) 1.0% strain amplitude at 600 °C; (**e**,**f**) 0.15% strain amplitude at 800 °C; (**g**,**h**) 0.6% strain amplitude at 800 °C; (**i**) instantaneous fault zone at 600 °C; and (**j**) instantaneous fault zone at 800 °C.

The fracture surfaces of 316H stainless steel subjected to low-cycle fatigue (LCF) testing are presented in Figure 12. Distinct radial patterns and fatigue striations are observed, indicating a predominantly fatigue-driven fracture mode. As the crack propagates, the spacing of fatigue striations gradually increases, accompanied by a change in the smoothness of the fracture surface. The fracture surface can be categorized into three regions: the crack initiation zone, propagation zone, and instantaneous fault zone. Fatigue cracks consistently originate at the specimen surface. For specimens tested at low strain amplitudes, a single-crack initiation site is observed, accompanied by radial patterns and a limited number of secondary cracks. In contrast, specimens tested at high strain amplitudes exhibit multiple-crack initiation sites and a higher density of secondary cracks. Within the crack propagation zone, well-defined fatigue striations are evident, with their spacing progressively increasing as the crack extends. The final rupture zone at 600 °C is relatively small, accounting for approximately 1/4 of the total fracture area, and is characterized by dimple structures; whereas at 800 °C, this zone expands to 1/2 of the fracture area and displays a mixed fracture morphology comprising intergranular facets and dimple structures.

**Figure 13:**
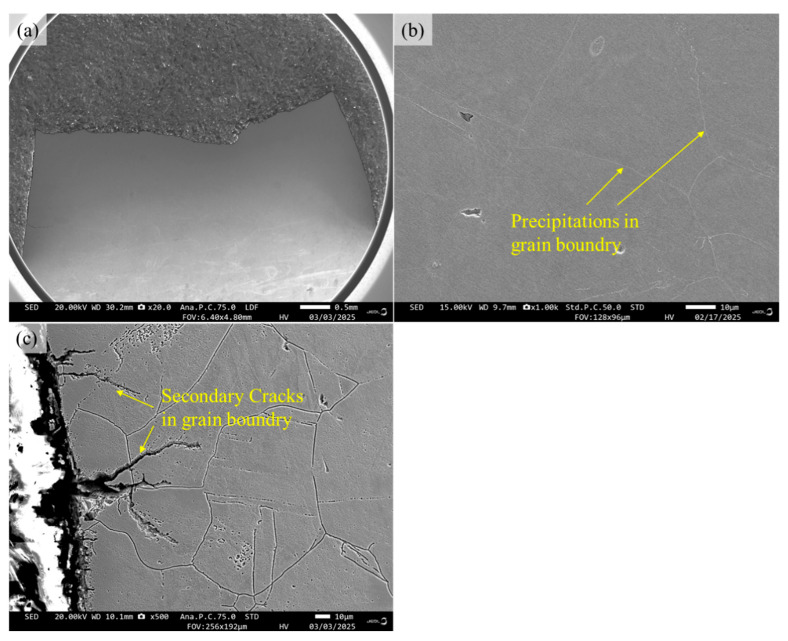
Cross-section view of 316H stainless steel at 600 °C with 1.0% strain amplitude: (**a**) macroscopic morphology, (**b**) microscopic morphology, and (**c**) details of secondary cracks.

**Figure 14:**
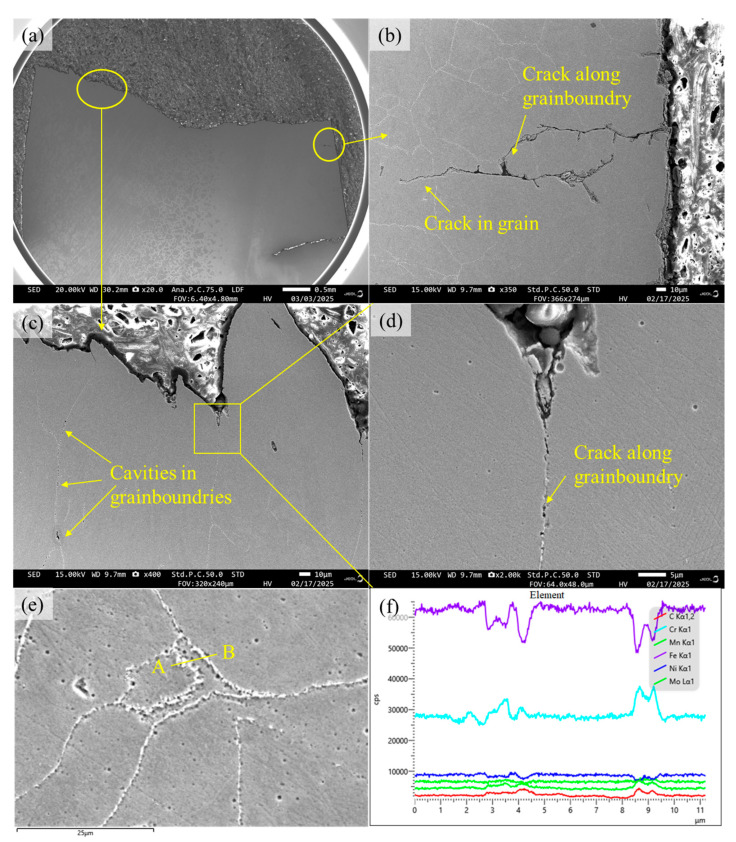
Cross-section view of 316H stainless steel at 800 °C with 0.6% strain amplitude: (**a**) macroscopic morphology, (**b**) details of secondary cracks, (**c**) grain boundaries in instantaneous fault zone, (**d**) details of grain boundaries in instantaneous fault zone, (**e**) precipitation on grain boundaries, and (**f**) elemental variations from point A to point B.

The cross-sectional microstructures near the fracture surfaces of 316H stainless steel at 600 °C and 800 °C temperatures are illustrated in Figure 13 and Figure 14. Cross-sectional microstructural analysis near the fracture surface reveals distinct crack propagation behaviors at 600 °C and 800 °C. After fatigue testing at 600 °C, secondary cracks predominantly propagate in a transgranular manner, with no significant evidence of intergranular cracking. A limited number of precipitates are observed along grain boundaries. In contrast, after 800 °C testing, secondary cracks exhibit a mixed propagation mode, combining both transgranular and intergranular paths. In the vicinity of the fatigue fracture surface, partial cavities are observed along the grain boundaries, accompanied by a significant presence of precipitates. Elemental scanning was performed from point A to point B in Figure 14e. Analysis of the elemental content within the precipitate revealed lower concentrations of Fe and Ni, along with elevated levels of Cr, C, and Mo/Mn. Based on the common types of precipitates formed in 316H at elevated temperatures, it is inferred that this precipitate is M_23_C_6_ [11].

Based on the empirical relationship (Equation (1)) between fracture toughness and the volume fraction of the second phase (*V_f_*) in copper alloys, it can be indicated that an increase in the volume fraction of second-phase particles leads to a deterioration in plastic indicators and enhanced material brittleness [37]. Consequently, at 800 °C, when a large number of coarsened M_23_C_6_ second-phase particles accumulate at grain boundaries, the grain boundary cohesive energy is reduced, thereby promoting intergranular fracture. Simultaneously, vacancy clustering at grain boundaries becomes notably pronounced at 800 °C. It can therefore be inferred that the fatigue fracture mechanism of 316H stainless steel at 800 °C results from the combined action of the two aforementioned factors. This indicates that both grain boundary strengthening and second-phase strengthening undergo rapid degradation within a relatively short duration at 800 °C, ultimately leading to a significant reduction in the fatigue performance at this temperature.

(1) $$\varepsilon_{f}^{\prime }=\frac{\lambda \left(1-V_{f}\right)}{V_{f}}$$

### 3.4. Discussion

Analysis of the Masing behavior under varying strain amplitudes reveals that a strain amplitude of approximately 0.6% represents a critical threshold for the occurrence of Masing behavior. The occurrence of Masing and non-Masing behaviors in materials is often closely associated with the evolution of dislocation structures [38,39,40,41]. The relevant literature [11] indicates that, at a low strain amplitude domain (0.2%~0.6%), the dislocation veins are observed in 316H. On the contrary, at the high strain amplitude domain (0.6%~1.0%), the dislocation cells are very intact, and the evolution of dislocation configuration from veins into cells corresponds to the transition from non-Masing behavior of 316H SS at the low strain amplitude domain to Masing behavior at the high strain amplitude domain. In this research, similar behavior is also observed at both 600 °C and 800 °C.

The dynamic strain aging (DSA) phenomenon observed with an increasing strain amplitude (Figure 9) and number of cycles (Figure 10) during strain-controlled fatigue further indicates that, under large strains and progressively accumulated fatigue damage, the dislocation distribution within the material microstructure likely undergoes gradually intensified interactions with both the matrix and solute atoms. This behavior resembles the mechanisms reported in the literature for inducing Masing behavior. It can therefore be inferred that the DSA phenomenon is also likely attributable to the gradual proliferation, entanglement, and formation of dislocation veins and cells during the fatigue process.

### 3.5. Fatigue Life Prediction

The Manson–Coffin equation [42,43] is widely employed for fatigue life prediction. This approach decomposes the total strain into elastic and plastic components, which are then fitted using logarithmic relationships. The relationship can be expressed as follows:

(2) $$\frac{∆\varepsilon_{e}}{2}=\frac{\sigma_{f}^{\prime }}{E}{\left({2N}_{f}\right)}^{b}$$

(3) $$\frac{∆\varepsilon_{p}}{2}={\varepsilon_{f}^{\prime }\left({2N}_{f}\right)}^{c}$$

(4) $$\frac{∆\varepsilon_{t}}{2}={\frac{\sigma_{f}^{\prime }}{E}{\left({2N}_{f}\right)}^{b}+\varepsilon_{f}^{\prime }\left({2N}_{f}\right)}^{c}$$

(5) $$\frac{∆\sigma }{2}={k^{\prime }\left({∆\varepsilon }_{p}/2\right)}^{n^{\prime }}$$

Among them, ${∆\varepsilon }_{e}$ is the elastic strain range, ${∆\varepsilon }_{p}$ is the plastic strain range, and ${∆\varepsilon }_{t}$ is the total strain range. $\sigma_{f}^{\prime }$ is the fatigue strength coefficient, *b* is the fatigue strength index, $\varepsilon_{f}^{\prime }$ is the fatigue ductility coefficient, *c* is the fatigue ductility index, and *E* is the half-life elastic modulus. Based on Equations (2) and (3), the measured elastic and plastic strains are plotted against the number of reversals to failure (2*N_f_*) on a double logarithmic scale. The resulting fitting parameters yield Equation (3), which represents the Manson–Coffin equation.

Furthermore, based on Equation (4), linear fitting is performed on the double logarithmic plot of ∆σ/2 versus ${∆\varepsilon }_{p}/2$, enabling the determination of the cyclic strength coefficient (*k’*) and the cyclic strain hardening exponent (*n’*) for low-cycle fatigue behavior.

The low-cycle fatigue fitting parameters for 316H stainless steel at temperatures ranging from 600 °C to 800 °C, calculated using the aforementioned methodology, are summarized in Table 5 [20].

The fatigue life was assessed using a plastic strain energy approach [34], expressed as $W_{p}=CN_{f}^{a}$. Where *N_f_* represents the fatigue life, *C* is the fitting coefficient, and *Wp* denotes the plastic strain energy per unit volume. The calculation involves plotting the stress–strain hysteresis loop at the stabilized cycle in the load–strain coordinate system, computing the area enclosed by the hysteresis loop, and normalizing it by the specimen’s cross-sectional area to obtain the plastic strain energy per unit volume. This energy parameter is then fitted to the fatigue life using a power-law relationship, yielding the corresponding *Wp*-*N_f_* correlation.

The relationship between the plastic strain energy per unit volume (*Wp*) and fatigue life (*N_f_*) at the tested temperatures is derived as follows:

At 600 °C, *N_f_* = 10,068.2466*W_p_*^−1.7286^, R^2^ = 0.9543;

At 650 °C, *N_f_* = 5674.3003*W_p_*^−1.9560^, R^2^ = 0.9129;

At 800 °C, *N_f_* = 2037.1118*W_p_*^−1.5875^, R^2^ = 0.9671.

A three-parameter method [2,44] was employed to fit the strain–life relationship, expressed as $N_{f}{\left(\varepsilon_{max}-\varepsilon_{0}\right)}^{m}=C$, with its logarithmic form given by $\mathrm{lg}N_{f}=B_{1}+B_{2}\mathrm{lg}\left(\varepsilon_{max}-B_{3}\right)$. The low-cycle fatigue life at various temperatures was fitted using this approach, yielding the following results:

At 600 °C, lg*N* = 2.3867 − 1.6840 × lg(ε − 0.165);

At 650 °C, lg*N* = 2.1889 − 1.5767 × lg(ε − 0.174);

At 800 °C, lg*N* = 2.2424 − 1.3883 × lg(ε − 0.123).

Where the unit of strain ε is%.

**Figure 15:**
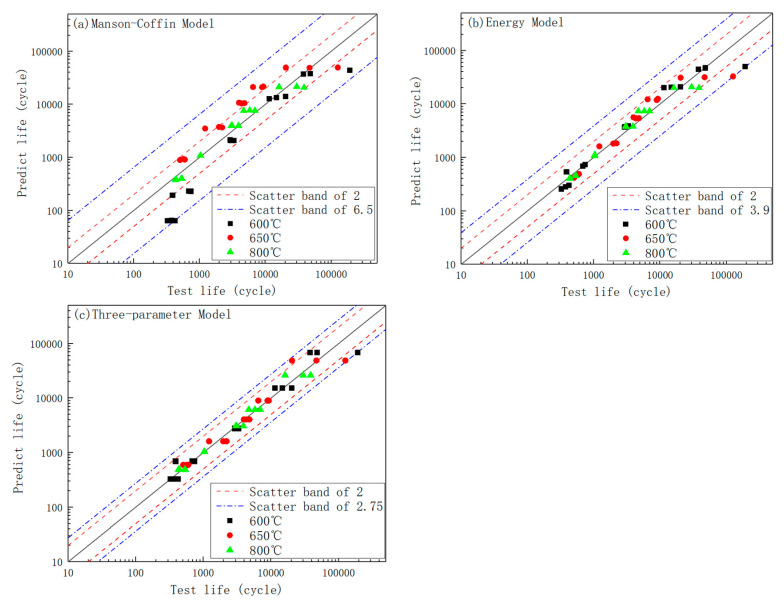
Comparison of fatigue life prediction results at (**a**) 600 °C, (**b**) 650 °C, and (**c**) 800 °C.

A comparison between the predicted and experimentally measured fatigue lives using the three models is illustrated in Figure 15. Although the Manson–Coffin equation is widely utilized, it exhibits limitations when applied to 316H stainless steel at elevated temperatures. Specifically, it underestimates the fatigue life at 600 °C, overestimates the life at 650 °C, and provides relatively accurate predictions at 800 °C, with a maximum error band of 6.5 times the actual life. In contrast, the energy-based approach demonstrates superior performance, yielding a maximum error band of 3.9 times the actual life, with no significant temperature-dependent discrepancies. The three-parameter method achieves the best performance among the three approaches, with a maximum error band of 2.75 times the actual life and no notable variations across different temperatures. Although the maximum error exceeds a factor of two, this is partially attributed to the inherent scatter in experimental data, particularly in the low-strain regime. In conclusion, the three-parameter method offers the most accurate prediction of high-temperature low-cycle fatigue life for 316H stainless steel.

## 4. Conclusions

1. The elastic modulus within the temperature range from room temperature to 800 °C exhibits a linear variation and remains relatively close to that of pure iron, indicating that the modulus of 316H stainless steel is predominantly governed by the matrix. The tensile strength decreases significantly by approximately 50%, while the plastic indicators initially decline and subsequently increase. Specifically, the elongation after fracture at 800 °C shows a 67% increase compared to the minimum value observed at 600 °C. Correspondingly, the tensile fracture microstructure at 800 °C displays a substantial number of voids, suggesting a pronounced vacancy diffusion-dominated failure mechanism at this temperature.
2. Fatigue life decreases with increasing temperature. At 600–800 °C, Masing behavior emerges at strain amplitudes above approximately 0.6%; dynamic strain aging (DSA) phenomena are observed at relatively higher strain amplitudes and higher fatigue cycles. The cyclic hardening rate is defined as the ratio of the increase in the stress amplitude to the increase in the number of cycles. The cyclic hardening rate increases with the temperature. At a given temperature, it approximately follows a power-law relationship with the strain amplitude.
3. At 600–800 °C, fatigue cracks initiate at the specimen surface, with single-crack initiation dominating at lower strain amplitudes and multiple-crack initiation prevailing at higher strain amplitudes, accompanied by an increased density of secondary cracks. At 600 °C, the final rupture zone is primarily characterized by ductile fracture, whereas at 800 °C, the rupture zone exhibits pronounced intergranular fracture. This transition is attributed to grain boundary weakening caused by the synergistic effects of elevated temperature and the accelerated coarsening of M_23_C_6_ precipitates.
4. For the assessment of fitting of fatigue life, the three-parameter equation demonstrates superior performance compared to the plastic strain energy approach and the Coffin–Manson equation, making it particularly suitable for predicting the high-temperature low-cycle fatigue life of 316H stainless steel under 600–800 °C conditions investigated in this study.

## Figures and Tables

**Table 1:** Chemical compositions of 316H steel (wt./%).

Element	C	Cr	Ni	Mo	Mn	S	P	Si	Co	Fe
Content	0.046	17.32	12.20	2.48	1.82	<0.0010	0.0094	0.41	<0.01	Bal.

**Table 2:** The total strain amplitude versus number of reversals to failure results for 316H stainless steel under high-temperature fatigue test (strain ratio R = −1).

600 °C		650 °C		800 °C	
Strain Amplitude, %	2*N_f_*, Cycle	Strain Amplitude, %	2*N_f_*, Cycle	Strain Amplitude, %	2*N_f_*, Cycle
0.4	5956	0.2	93,824	0.6	1082
0.2	95,636	0.3	8650	0.25	6046
0.2	75,140	0.25	12,980	0.25	7804
0.7	1474	0.25	17,782	0.25	6224
0.25	22,702	0.6	1196	0.15	32,300
1.0	644	0.25	18,576	0.15	59,844
0.4	5806	0.2	40,836	0.15	77,232
1.0	748	0.6	1148	0.2	13,850
0.25	40,486	0.4	3944	0.2	9382
0.7	772	0.3	7938	0.2	11,548
1.0	846	0.3	9602	0.6	858
0.25	29,312	0.6	1008	0.6	886
0.4	6598	0.2	251,570	0.6	1058
0.7	1362	0.4	4420	0.4	2076
0.2	379,018	0.4	2430	0.4	2054
				0.4	2056

**Table 3:** Cyclic hardening rate of 316H stainless steel at 600 °C~800 °C.

600 °C		650 °C		800 °C	
Strain Amplitude %	Cyclic Hardening Rate MPa/Cycle	Strain Amplitude %	Cyclic Hardening Rate MPa/Cycle	Strain Amplitude %	Cyclic Hardening Rate MPa/Cycle
1	11.66	0.6	9.10	0.6	21.46
0.7	7.51	0.4	3.60	0.4	8.37
0.4	1.23	0.3	1.06	0.25	4.25
0.25	0.34	0.25	0.47	0.2	2.05
0.2	0.16	0.2	0.18	0.15	0.99

**Table 4:** Fitting results of cyclic hardening rate of 316H stainless steel at 600 °C~800 °C.

Temperature °C	a	b	R
600	70,379	1.88217	0.9588
650	1.12862 × 10^7^	2.74051	0.9845
800	1.09182 × 10^6^	2.11961	0.9937

**Table 5:** Low-cycle fatigue fitting parameters of 316H stainless steel at 600–800 °C.

Fitting Parameters	600 °C	650 °C	800 °C
Fatigue strength coefficient $\sigma_{f}^{\prime }$	1053	632	452
Fatigue strength exponent *b*	−0.1577	−0.1118	−0.1310
Fatigue ductility coefficient $\varepsilon_{f}^{\prime }$	0.0847	0.0378	0.1246
Fatigue ductility index *c*	−0.4097	−0.3485	−0.4983
Cyclic strength coefficient $k^{\prime }$	1345	1273	427
Cyclic strain hardening exponent $n^{\prime }$	0.2708	0.2663	0.1683
Coefficient of determination R^2^	0.9479	0.8988	0.9630

## Data Availability

The original contributions presented in this study are included in the article. Further inquiries can be directed to the corresponding author.

## References

[1] Hong S., Lee K., Lee S. (2025). Dynamic strain aging effect on the fatigue resistance of type 316L stainless steel. Int. J. Fatigue.

[2] Ding K., Tang Z., He X., Wang X., He J. (2024). Low cycle fatigue characteristics and life prediction of 316LN austenitic stainless steel. Prog. Nat. Sci.-Mater. Int..

[3] EPRI (2019). Program on Technology Innovation: Materials Properties Assessment and Gap Analysis for Lead-Cooled Fast Reactors.

[4] Forsberg C. (2005). The advanced high-temperature reactor: High-temperature fuel, liquid salt coolant, liquid-metal-reactor plant. Prog. Nucl. Energy.

[5] Hahn D., Chang J., Kim Y., Kim Y., Lee C.B., Kim S., Lee J., Ha K., Kim B., Lee Y. (2009). Advanced SFR design concepts and R&D activities. Nucl. Eng. Technol..

[6] Aoto K., Uto N., Sakamoto Y., Ito T., Toda M., Kotake S. (2011). Design study and R&D progress on Japan sodium-cooled fast reactor. J. Nucl. Sci. Technol..

[7] Lee K.L., Ha K.S., Jeong J.H., Choi C.W., Jeong T., Ahn S.J., Lee S.W., Chang W.P., Kang S.H., Yoo J. (2016). A preliminary safety analysis for the prototype gen IV sodium-cooled fast reactor. Nucl. Eng. Technol..

[8] Xu H. (2008). Structural materials in fast reactor. Chin. J. Nucl. Sci. Eng..

[9] Li X. (2018). Study on Ferrite and Grain Size of Nuclear Power 316H Austenitic Stainless Steel. Master’s Thesis.

[10] ASME (2007). ASME Boiler and Pressure Vessel Code.

[11] Yang J., Li B., Wang K., Zhao L., Xu L., Chen X. (2024). Cyclic deformation behavior and damage mechanism of 316H stainless steel under low-cycle fatigue loading at 650 °C. Int. J. Fatigue.

[12] Xu L., Bao F., Zhao L., Han Y., Jing H., Yu H., Gong X. (2021). Characterizing microstructural evolution and low cycle fatigue behavior of 316H austenitic steel at high-temperatures. J. Nucl. Mater..

[13] Zhao L., Qi X., Xu L., Han Y., Jing H., Song K. (2021). Tensile mechanical properties, deformation mechanisms, fatigue behaviour and fatigue life of 316H austenitic stainless steel: Effects of grain size. Fatigue Fract. Eng. Mater. Struct..

[14] (2017). Specification for Forged or Rolled Alloy and Stainless Steel Pipe Flanges, Forged Fittings, and Valves and Parts for High-Temperature Service.

[15] (1989). Methods for Chemical Analysis of Iron, Steel and Alloy.

[16] (2010). Metallic Materials—Tensile Testing—Part 1: Method of Test at Room Temperature.

[17] (2015). Metallic Materials—Tensile Testing—Part 2: Method of Test at Elevated Temperature.

[18] (2008). Metallic Materials—Determination of Modulus of Elasticity and Poisson’s Ratio.

[19] (2008). The Test Method for Axial Loading Constant-Amplitude Low-Cycle Fatigue of Metallic Materials.

[20] Zhang H., Zhu M., Sun X., He D., Wang Q., Su D., Li N., Zeng C., He X. (2021). Research on Fundamental Characteristics of Nuclear Grade 316H Stainless Steel at Ultra High Temperature. Nucl. Power Eng..

[21] Richter F. (1973). Die wichtigsten physikalischen Eigenschaften von 52 Eisenwerkstoffen. Stahleisen-Sonderberichte.

[22] Hougardy H.P. (1984). Werkstoffkunde Stahl Band 1: Grundlage.

[23] Richter F. (1983). Physikalische Eigenschaften Von Stählen und Ihre Temperaturabhängigkeit: Polynome und Graphische Darstellungen.

[24] Richter F., Hemminger W., Hanitzsch E. (1991). Physical properties in the temperature range from–180 to 400 °C of nickel alloy steels for low-temperature applications. Steel Res..

[25] Mikio F., Sanpei A. (1993). Elastic moduli and internal friction of low carbon and stainless steels as a function of temperature. ISIJ Int..

[26] Fink K., Richter F., Lotter U., Schrecke K. (1970). Physikalische Eigenschaften von Stählen, insbesondere von warmfesten Stählen. Thyssenforschung.

[27] Lee S.S., Min U.S., Ahn B., Yoo S.H. (1998). Elastic constants determination of thin cold-rolled stainless steels by dynamic elastic modulus measurements. J. Mater. Sci..

[28] Zhang J. (2007). High-Temperature Deformation and Fracture of Materials.

[29] Lv X., Chen S., Rong L. (2024). Absence of dynamic strain aging induced by precipitation in 316 austenitic stainless steel during thermal aging at 750 °C. Mater. Charact..

[30] Cottrell A.H., Hunter S.C., Nabarro F.R.N. (1953). CXI. Electrical interaction of a dislocation and a solute atom. Lond. Edinb. Dublin Philos. Mag. J. Sci..

[31] McCormigk P.G. (1972). A model for the Portevin-Le Chatelier effect in substitutional alloys. Acta Metall..

[32] van den Beukel A. (1975). Theory of the effect of dynamic strain aging on mechanical properties. Phys. Status Solidi (A).

[33] Hong S.G., Lee S.B. (2004). The tensile and low-cycle fatigue behavior of cold worked 316L stainless steel: Influence of dynamic strain aging. Int. J. Fatigue.

[34] Schoeck G. (1984). The Portevin-Le Chatelier effect. A kinetic theory. Acta Metall..

[35] Macek W., Branco R., de Jesus J., Costa J.D., Zhu S.P., Nejad R.M., Gryguć A. (2024). Strain energy density and entire fracture surface parameters relationship for LCF life prediction of additively manufactured 18Ni300 steel. Int. J. Damage Mech..

[36] Chan K.S., Miller A.K. Fatigmod: A unified phenomenological model for predicting fatigue crack initiation and propagation. Proceedings of the ASME International Conference on Advances in Life Prediction Methods.

[37] Zhong Q., Fractography Z.Z. (2006). Fractography.

[38] Yadav S.S., Roy S.C., Goyal S. (2023). A comprehensive review and analysis of Masing/nonMasing behavior of materials under fatigue. Fatigue Fract. Eng. Mater. Struct..

[39] Plumtree A., Abdel-Raouf H.A. (2001). Cyclic stress–strain response and substructure. Int. J. Fatigue.

[40] Verma P., Srinivas N.C.S., Singh V. (2018). Low cycle fatigue behavior of modified 9Cr-1Mo steel at 300 °C. Mater. Sci. Eng. A.

[41] Watanabe E., Asao T., Toda M., Yoshida M., Horibe S. (2013). Relationship between masing behavior and dislocation structure of AISI 1025 under different stress ratios in cyclic deformation. Mater. Sci. Eng. A.

[42] Manson S.S. (1965). Fatigue: A complex subject—Some simple approximations. Exp. Mech..

[43] Coffin L.F. (1954). A study of the effects of cyclic thermal stresses on a ductile metal. Trans. ASME.

[44] (2020). Data Reduction and Presentation of Mechanical Property for Metallic Materials.

